# Comparing in vivo and ex vivo features of amelanotic melanoma using reflectance confocal microscopy and line-field confocal optical coherence tomography

**DOI:** 10.1016/j.jdcr.2024.11.022

**Published:** 2024-11-30

**Authors:** Isabella J. Tan, Noah Musolff, Madeline Tchack, Bianca Sanabria, Thu M. Truong, Victoria Caetano, Shazli Razi, Babar Rao

**Affiliations:** a Rutgers Robert Wood Johnson Medical School, The State University of New Jersey, Piscataway, New Jersey; b Rao Dermatology, Atlantic Highlands, New Jersey; c Center for Dermatology, Rutgers Robert Wood Johnson Medical School, Somerset, New Jersey; d Department of Internal Medicine, Overlook Medical Center, Summit, New Jersey; e Department of Internal Medicine, Jersey Shore University Medical Center, Neptune, New Jersey; f Deparment of Dermatology, Weill Cornell Medicine, New York, New York

**Keywords:** amelanotic, line-field confocal optical coherence tomography, melanoma, reflectance confocal microscopy

## Introduction

Amelanotic melanoma, a pigment-lacking melanoma variant, is relatively rare, constituting 2% to 8% of melanoma cases.^1^ It can affect individuals of any age and gender, often occurring in sun-exposed areas. Reflectance confocal microscopy (RCM) is commonly used for diagnosing basal cell carcinoma (BCC), squamous cell carcinoma, and pigmented lesions.^2^ However, its depth of visualization is limited to superficial skin layers.^3^ For amelanotic melanoma, RCM typically reveals epidermal atypia with dermoepidermal junction (DEJ) disarray.^3^ One systematic review found a 12.1% false negative rate for RCM in distinguishing amelanotic melanoma from other nonmelanocytic conditions.^2^ In contrast, line-field confocal optical coherence tomography (LC-OCT) provides both en face and cross-sectional views, allowing for deeper tissue visualization. Herein, we present a case of amelanotic melanoma with in vivo imaging and histopathologic correlation.

## Clinical presentation

**Fig 1:**
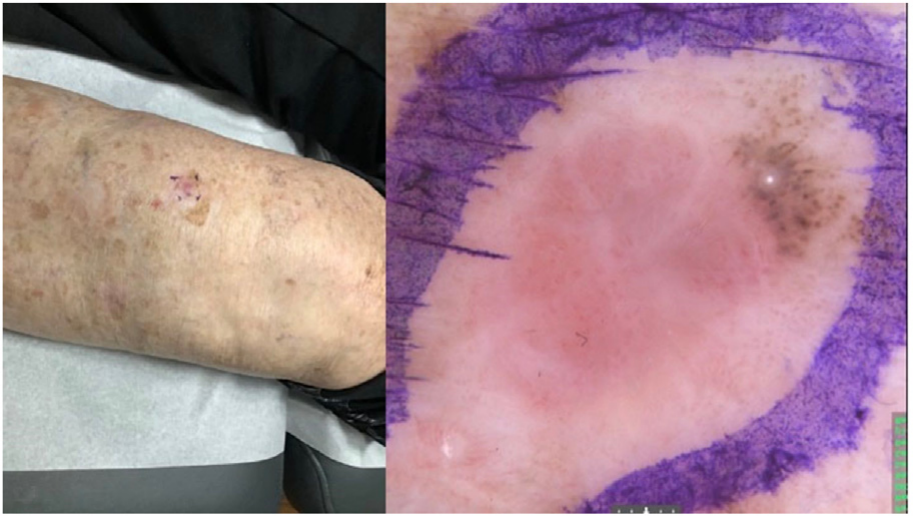
Clinical photo and dermoscopy image.

An 88-year-old woman presented with a progressively enlarging tumor on her right lower leg, appearing as a slightly red, 1 cm papule, with surrounding pigmentation (Fig 1). Dermoscopy revealed a light pink, asymmetrical papule with irregular clusters of brown pigmented globules at the periphery (Fig 1). The presence of polymorphic vessels, milky pink areas, and white structureless areas resembled typical BCC features, leading to an initial suspicion of BCC. However, due to the atypical vascular patterns and absence of classic pigmentation, RCM and LC-OCT were performed for further in vivo evaluation.

## RCM

**Fig 2:**
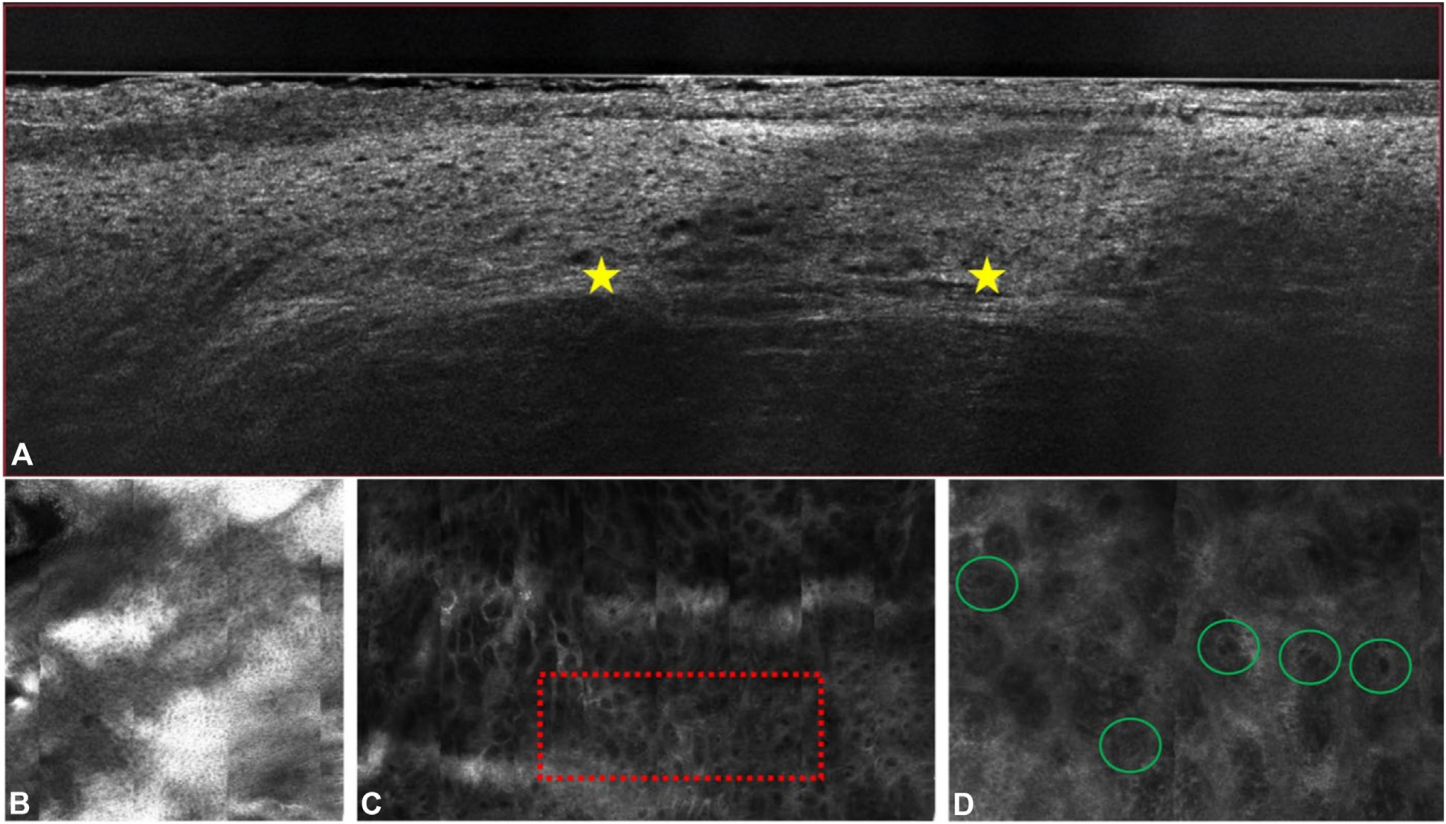
Line-field confocal optical coherence tomography (LC-OCT) compared to reflectance confocal microscopy (RCM). **A,** LC-OCT: Vertical view shows disruption of the basal layer, loss of rete ridges, and deep bright fibers (*yellow stars*). **B,** RCM: Epidermis demonstrates atypical honeycombing and architectural disarray. **C,** RCM: Mosaic-map overview of dermoepithelial layer with area of interest (*red dotted box*). **D,** RCM: View of *red dotted box* seen in 2C; presence of cluster of hyporeflective nucleated cells (*green circles*).

RCM demonstrated atypical honeycombing with architectural disarray in the epidermis, the presence of atypical ovoid pagetoid cells at the DEJ, and clusters of hyporeflective nucleated cells in the dermis (Fig 2), consistent with previous findings of melanoma on RCM.^4^

## LC-OCT

**Fig 3:**
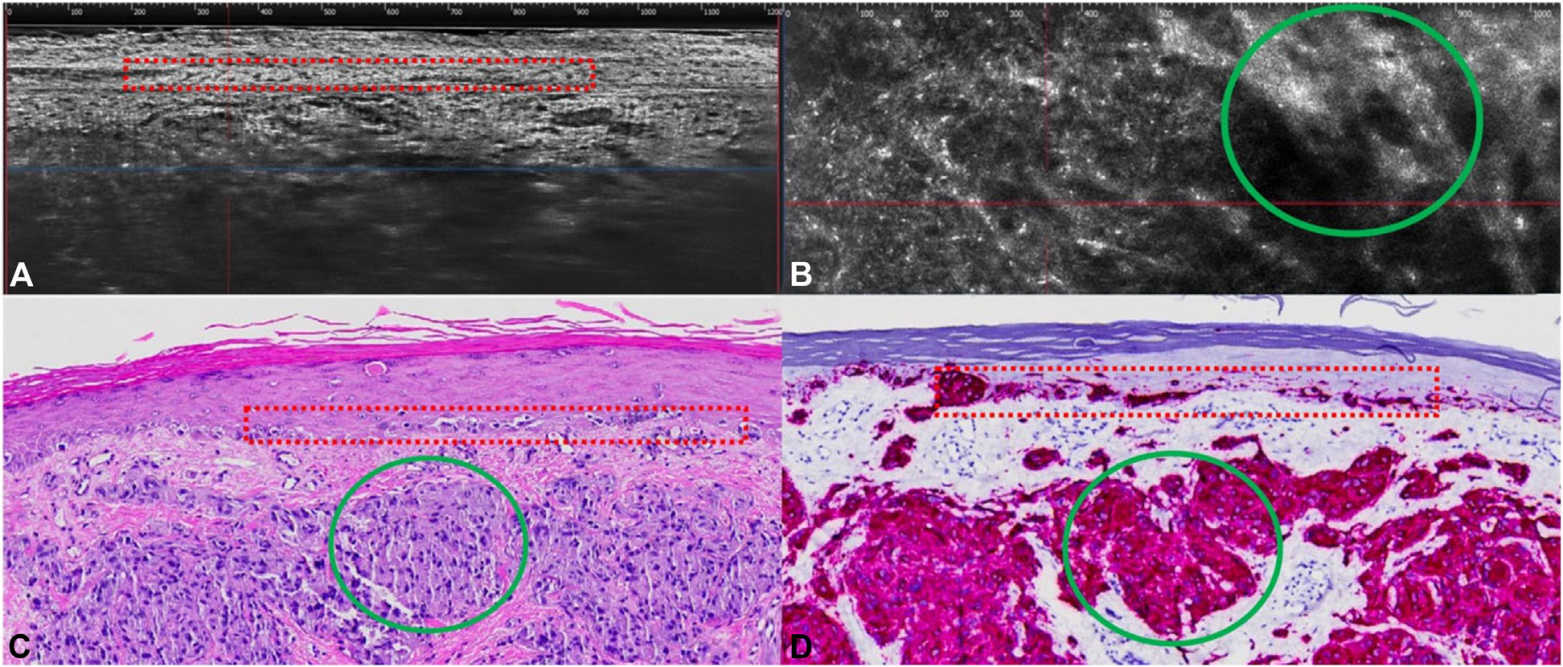
Line-field confocal optical coherence tomography (LC-OCT) compared to histology. **A,** LC-OCT: Vertical view shows disruption of basal layer and loss of rete ridges (*red dotted box*). **B,** LC-OCT: Horizontal view at the level of the papillary dermis, large dermal clusters of hyper-reflective cells with bright fibers (*green circle*). **C,** Hematoxylin and eosin (H&E) at 400× showing melanocyte nests of varying sizes and shapes, unevenly distributed at the dermal-epidermal junction (*red dotted box*) and dermal nests (*green circle*). **D,** MART staining at 400× showing melanocyte nests in varying sizes and shapes, unevenly distributed at the dermal-epidermal junction (*red dotted box*) and dermal nests (*green circle*).

LC-OCT revealed disruption of the basal layer and loss of rete ridges, with atypical pagetoid cells exhibiting large nuclei in the epidermis (Fig 3). Within the dermis, there were clusters of hyper-reflective cells embedded in bright, thickened fibers.

## Histopathology

Histopathology showed a poorly demarcated lesion composed of several atypical melanocytes with large, hyperchromatic, pleomorphic nuclei, and abundant cytoplasm in the epidermis. At the DEJ and within the dermis, several clusters of atypical melanocytes were found, extending up to 1.0 mm from the granular layer and surrounded by a fibrotic dermis. Immunohistochemical stains, including MART-1, S100, and SOX-10, were positive, with focal positivity for HMB45, confirming the diagnosis of melanoma (Fig 3).

## Key message

This case highlights the diagnostic challenges of amelanotic melanoma, which can resemble BCC, benign nevus, clear cell acanthoma, and other conditions. The gold standard for patient safety is histological evaluation. Before performing a biopsy, we utilized RCM and LC-OCT to assess their diagnostic potential. Although these technologies are not universally accessible due to cost, limited availability, and the need for specialized training, combining histopathological, dermoscopic, and noninvasive imaging techniques may enhance diagnostic confidence for amelanotic melanoma. While RCM showed features of melanoma,^4^ including pagetoid cells, LC-OCT provided insights regarding depth and structural aberrations, including hyper-reflective atypical cellular clusters, rete ridge loss, and fibrosis. Further research is needed to expand the utility of LC-OCT in clinical practice.

## Conflicts of interest

Dr Rao is a speaker for Incyte. Authors Tan, Tchack, Sanabria, Caetano, and Drs Musolff, Truong, and Razi have no conflicts of interest to declare.
